# The Chimp Genome Reveals a Retroviral Invasion in Primate Evolution

**DOI:** 10.1371/journal.pbio.0030126

**Published:** 2005-03-01

It's been known for a long time that only 2%–3% of human DNA codes for proteins. Much of the rest of our genomes—often referred to as junk DNA—consists of retroelements: genomic elements that are transcribed into RNA, reverse-transcribed into DNA, and then reinserted into a new spot in the genome. Human endogenous retroviruses make up one class of these retroelements. Retroviruses can insinuate themselves into the host's DNA in either soma (nonreproductive cells) or the germline (sperm or egg).

If the virus invades a nonreproductive cell, infection may spread, but viral DNA will die with the host. A retrovirus is called endogenous when it invades the germline and gets passed on to offspring. Because endogenous retroviruses can alter gene function and genome structure, they can influence the evolution of their host species. Over 8% of our genome is made of these infectious remnants—infections that scientists believe occurred before Old World and New World monkeys diverged (25–35 million years ago).

In a new study, Evan Eichler and colleagues scanned finished chimpanzee genome sequence for endogenous retroviral elements, and found one (called PTERV1) that does not occur in humans. Searching the genomes of a subset of apes and monkeys revealed that the retrovirus had integrated into the germline of African great apes and Old World monkeys—but did not infect humans and Asian apes (orangutan, siamang, and gibbon). This undermines the notion that an ancient infection invaded an ancestral primate lineage, since great apes (including humans) share a common ancestor with Old World monkeys.

Eichler and colleagues found over 100 copies of PTERV1 in each African ape (chimp and gorilla) and Old World monkey (baboon and macaque) species. The authors compared the sites of viral integration in each of these primates and found that few if any of these insertion sites were shared among the primates. It appears therefore that the sequences have not been conserved from a common ancestor, but are specific to each lineage.

PTERV1 contains three structural genes—*gag*, *pol*, and *env*—and regulatory sequences called long terminal repeats (LTRs). To further explore the evolutionary history of the retroviral elements, the authors compared the sequences of *gag* and *pol*, as well as the LTR sequences, for each infected primate species. The sequence history, they discovered, did not comport with the established evolutionary history of the primates themselves. Divergence between macaque and baboon was significantly greater than between gorilla and chimp—even though slightly more evolutionary time separates gorilla and chimp than macaque and baboon.

When a retrovirus reproduces, identical copies of LTR sequences are created on either side of the retroviral element; the divergence of LTR sequences within a species can be used to estimate the age of an initial infection. Eichler and colleagues estimate that gorillas and chimps were infected about 3–4 million years ago, and baboon and macaque about 1.5 million years ago. The disconnect between the evolutionary history of the retrovirus and the primates, the authors conclude, could be explained if the Old World monkeys were infected by “several diverged viruses” while gorilla and chimpanzee were infected by a single, though unknown, source.[Fig pbio-0030126-g001]

**Figure pbio-0030126-g001:**
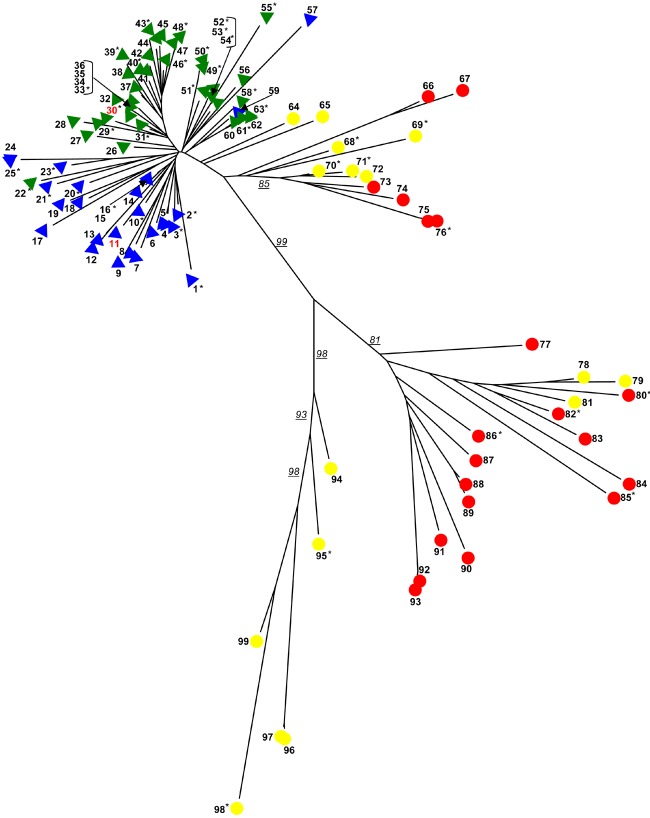
Phylogenetic tree of retroviral insertions in primates

As for how this retroviral infection bypassed orangutans and humans, the authors offer a number of possible scenarios but dismiss geographic isolation: even though Asian and African apes were mostly isolated during the Miocene era (spanning 24 to 5 million years ago), humans and African apes did overlap. It could be that African apes evolved a susceptibility to infection, for example, or that humans and Asian apes evolved resistance. A better understanding of the evolutionary history and population genetics of great apes will help identify the most likely scenarios. And knowing how these retroviral elements infiltrated some apes while sparing others could provide valuable insights into the process of evolution itself.

